# Mechanical Aortic Valve Dehiscence and Aortic Root Aneurysm: An Interesting Case With a Timely Diagnosis and Intervention

**DOI:** 10.7759/cureus.63432

**Published:** 2024-06-29

**Authors:** Hema Pamulapati, Pramod Janga, Siva Sagar Taduru, Ajay Kumar Kaja

**Affiliations:** 1 Cardiovascular Disease, University of Kansas Medical Center, Kansas City, USA; 2 Hospital Medicine, Atrium Health, Charlotte, USA; 3 Internal Medicine, Hays Medical Center, Hays, USA; 4 Cardiovascular Medicine, University of Kansas, Kansas City, USA; 5 Cardiology, Piedmont Heart Institute, Fayetteville, USA

**Keywords:** redo surgical aortic valve replacement, mechanical aortic valve prosthesis, aortic root replacement, aortic root dilation, endocarditis, aortic valve insufficiency, valve dehiscence, prosthetic heart valve

## Abstract

Prosthetic aortic valve dehiscence is a rare but potentially life-threatening complication that can occur after aortic valve replacement surgery. This condition occurs when the prosthetic valve becomes detached or dislodged from its original position leading to aortic valve regurgitation and congestive heart failure. The most common risk factors for prosthetic valve dehiscence include infective endocarditis, ascending aortic aneurysm, and severe calcification of the aortic valve. Ankylosing spondylitis, non-infectious aortitis, and accompanying vasculitis can also cause aortic valve dehiscence. Transthoracic echocardiography and transesophageal echocardiography usually reveal an unstable prosthesis with rocking motion and paravalvular regurgitation. Fluoroscopy and cardiac computed tomography (CT) are useful complementary tests, especially in patients with significant artifacts related to a valve prosthesis. Patients with prosthetic valve dehiscence and paravalvular regurgitation eventually develop heart failure and circulatory collapse. Timely diagnosis and early surgical intervention in these patients are crucial to achieve good long-term outcomes.

## Introduction

Prosthetic aortic valve dehiscence refers to the disintegration of suture material, leading to partial or complete detachment of the aortic valve prosthesis from the annulus. It is a rare and potentially fatal complication reported in 0.1-1.3% of patients who undergo aortic valve replacement [1]. The most common predisposing factors for dehiscence include previous endocarditis (12.2%), ascending aortic aneurysms (10.9%), degenerative regurgitation (7%), or extensive calcifications (6%) [2,3]. Co-existing vasculitis from Behcet's disease and ankylosing spondylitis have also been identified as the non-infectious etiology of prosthetic valve dehiscence [3,4]. The risk of prosthetic valve endocarditis is similar for both bioprosthetic and mechanical valves and has an incidence of 0.3% to 1.2% per patient-year [5,6]. Prior studies showed that the real pathophysiology of late dehiscence is often related to annular damage generated by the original infection rather than infection persistence. Clinical presentation varies from asymptomatic to abrupt heart failure, cardiogenic shock, and sudden death. Early diagnosis and treatment are key to preventing significant morbidity and mortality in these patients.

## Case presentation

**Figure 1:**
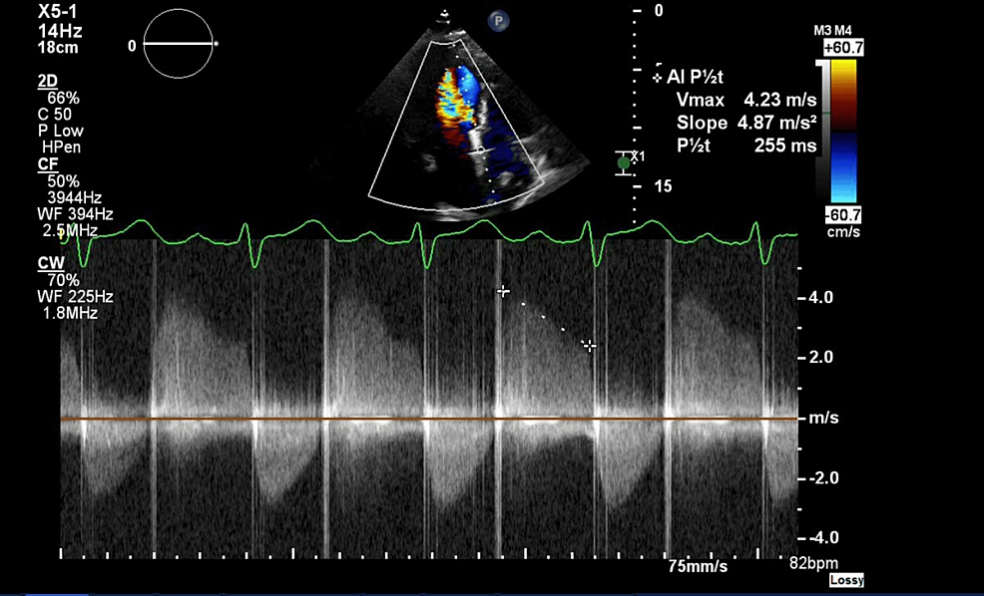
Spectral Doppler showing moderate to severe paravalvular aortic regurgitation

**Figure 2:**
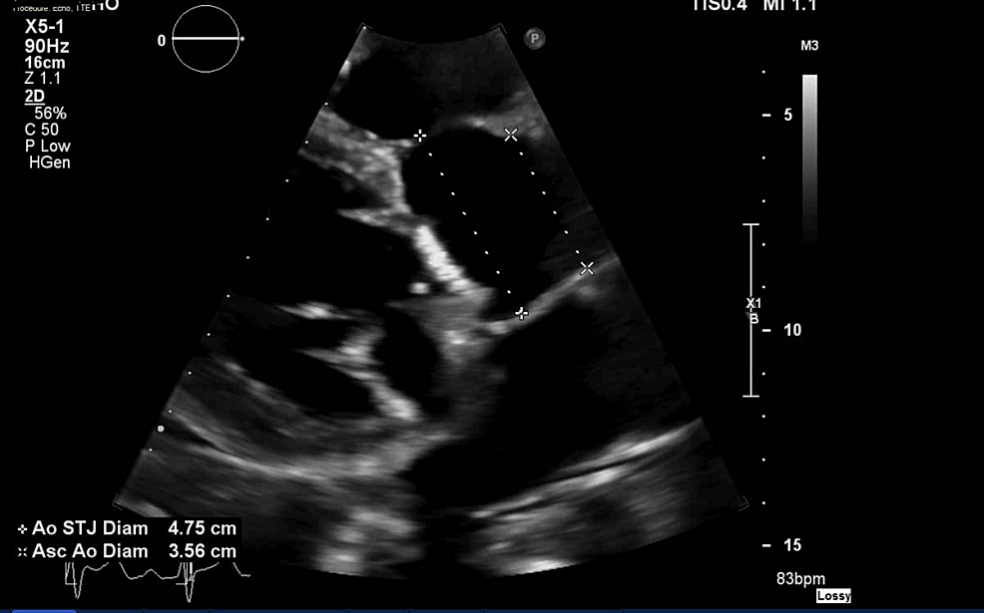
TTE showing a dilated aortic root, measuring 4.75 cm at the sinotubular junction TTE: transthoracic echocardiogram

We present a case of a 59-year-old female with mechanical aortic valve dehiscence and severe paravalvular regurgitation requiring urgent aortic valve replacement. She had a past medical history of hypertension, hyperlipidemia, paroxysmal atrial fibrillation, heart failure with preserved ejection fraction, severe aortic insufficiency status post aortic valve replacement, with a 21 mm St. Jude mechanical aortic valve (St. Jude Medical Inc, St. Paul, Minnesota, U.S.) in 8/2020. She was transferred to our hospital in 1/2023 with septic shock and a single blood culture positive for streptococcus group A bacteremia at an outside facility, acute renal failure, and multiple metabolic derangements. She received intravenous (IV) piperacillin and tazobactam prior to transfer and repeat blood cultures on admission showed no growth. Upon admission, she went into atrial fibrillation with rapid ventricular response in the setting of sepsis and converted back to sinus rhythm with the initiation of amiodarone. She was subsequently diagnosed with L1-L2 discitis and osteomyelitis. Later on, she underwent posterolateral fusion of L1-L2 with allograft. Transthoracic echocardiogram during the hospital stay showed normal left ventricular ejection fraction (LVEF) in the 55-60% range, normal right ventricular size and systolic function, normal bi-atrial size, and a St. Jude mechanical aortic valve functioning normally with peak velocity 2.19 m/s and mean gradient 10 mmHg. She was continued on IV antibiotics with IV ceftriaxone for four weeks post-discharge from the hospital. She presented for a follow-up visit four weeks after discharge from the hospital and complained of lightheadedness, weakness, and low blood pressure at home, requiring midodrine. She underwent a repeat echocardiogram, which showed normal LVEF in the 60% range, normal right ventricular size and contractility, and mechanical prosthesis in the aortic position with rocking motion suggesting prosthetic valve dehiscence with moderate to severe paravalvular regurgitation from the anterior margin (Videos 1-4) (Figure 1). The aortic root was dilated at 4.75 cm, compared to 3.7 cm on a prior echo (Figure 2).

**Video 1:** Parasternal long-axis view showing the rocking motion and dehiscence of the mechanical aortic valve

**Video 2:** Mechanical aortic valve dehiscence with para-valvular aortic regurgitation on color Doppler

**Video 3:** Apical five-chamber view showing prosthetic aortic valve dehiscence

**Video 4:** Apical five-chamber view with color Doppler showing paravalvular aortic regurgitation

She was referred to cardiothoracic surgery and underwent a CTA of the chest, which showed a dilated aortic root measuring 4.8 cm at the sinus of Valsalva without evidence of abscess or thoracic aortic dissection. She underwent a redo sternotomy with mechanical aortic valve replacement and aortic root replacement. She developed a complete heart block on postoperative day 2 and subsequently underwent pacemaker placement. She was discharged home on postoperative day 7. Surgical specimen culture and pathology did not reveal any evidence of infection. She continued follow-up with infectious diseases and was recommended to continue ceftriaxone for a total duration of four to six weeks postoperatively. Postoperative echocardiogram showed normal LVEF in the 55% range, normal right ventricular size and systolic function, pacemaker lead in right-sided chambers, and a well-seated mechanical aortic valve prosthesis that appeared to be functioning normally with a peak velocity of 2.5 m/s and a mean gradient of 15 mmHg. She has recovered well postoperatively and continues to do well.

## Discussion

Mechanical aortic valve dehiscence is a rare complication associated with significant morbidity and mortality. The most common cause of mechanical aortic valve dehiscence is infective endocarditis and it carries a mortality risk of about 25-30% [7]. Mechanical aortic valve endocarditis is relatively more common than mechanical mitral valve endocarditis, 0.27% vs 0.18% per patient-year [8]. Based on the timing, prosthetic valve endocarditis can be either early onset or late onset. Early onset usually happens within one year of valve replacement and is most frequently caused by Staphylococcus aureus and less commonly by coagulase-negative staphylococci. Late cases are usually community-acquired and caused by Streptococcus viridans or the HACEK (Haemophilus species, Aggregatibacter actinomycetemcomitans, Cardiobacterium hominis, Eikenella corrodens, and Kingella kingae) group of bacteria [9]. Over two-thirds of prosthetic valve endocarditis (PVE) happens in the year following valve replacement [9,10]. Due to inadequate endothelization, mechanical prostheses are more susceptible to early infection than bioprostheses [9,10]. On the other hand, because of the progressive degeneration of the leaflets, bioprostheses are more susceptible to late PVE [9,10]. However, the cumulative risk of PVE is similar for both types of valves [9]. Clinical presentation of PVE varies from fever, chills, malaise, septic shock, new or changed heart murmur, primary valve failure, heart failure, complications related to septic emboli, etc. Other signs like Osler's nodes, Janeway lesions, and Roth's spots are not common in these patients.

Echocardiography is the basic and most common imaging modality used for the diagnosis of PVE, according to current guidelines [9]. In cases where PVE is suspected, it is reasonable to repeat the echocardiographic examination after seven days. However, a diagnosis of prosthetic valve endocarditis can be difficult compared to native valve endocarditis on transthoracic echocardiography due to the presence of artifact/acoustic shadowing and body habitus. Transesophageal echocardiography (TEE) is the recommended diagnostic tool and provides better resolution with increased sensitivity (86-94% sensitivity) and specificity (88-100%) [7,10]. 3D TEE is a valuable tool in the assessment of PVE, as it provides clear and detailed anatomical visualization [7]. Echocardiographic findings include vegetation on the valvular structures (oscillating mass), abscess formation, and perforation or dehiscence of the prosthetic valve causing paravalvular regurgitation [7]. On echocardiography, prosthetic valve dehiscence is defined as a rocking motion that exceeds 15° in at least one plane, as was apparent in this case [10]. Cardiac CT and fluoroscopy are complementary imaging modalities in patients where visualization is limited due to shadowing from the mechanical valve [11]. The 18F-fluorodeoxyglucose positron emission tomography/computed tomography (18F-FDG PET/CT) scan is another supplementary test. Cardiac CT helps identify perivalvular complications like pseudoaneurysms and abscesses, assessing vascular structures and coronary anatomy. Microorganisms disrupt perivalvular tissue by invading the prosthetic ring [12]. This increases the risk of valvular dehiscence, abscess, pseudoaneurysm, or fistula formation.

The presence of complications like heart failure, large vegetation (>10 mm) with high embolization risk or valvular obstruction, abscess formation, valve dehiscence or dysfunction, non-HACEK gram-negative bacteria, persistently positive blood cultures, and staphylococcal and fungal bacteremia are indications for surgical treatment. Other hemodynamically stable prosthetic valve endocarditis patients can be managed with medical therapy similar to native valve endocarditis and require close monitoring during and after treatment [12]. Surgical treatment for PVE involves the debridement of infected tissue and the placement of a new valve in healthy tissue.

## Conclusions

Mechanical aortic valve dehiscence is a serious complication with a high mortality burden. Although prosthetic valve endocarditis is the most common cause, other noninfectious causes have been reported as well. Early diagnosis and treatment are crucial to prevent mortality in these patients. Multimodality imaging can be helpful in these patients for a more complete assessment and surgical planning. Close postoperative follow-up and a multidisciplinary team approach involving a cardiothoracic surgeon, cardiologist, and infectious disease specialist are beneficial in these patients. This case highlights the presence of mechanical aortic valve dehiscence in the setting of PVE, timely diagnosis, multidisciplinary approach, and successful outcome.
